# An examination of the causes, consequences, and policy responses to the migration of highly trained health personnel from the Philippines: the high cost of living/leaving—a mixed method study

**DOI:** 10.1186/s12960-017-0198-z

**Published:** 2017-03-31

**Authors:** Erlinda Castro-Palaganas, Denise L. Spitzer, Maria Midea M. Kabamalan, Marian C. Sanchez, Ruel Caricativo, Vivien Runnels, Ronald Labonté, Gail Tomblin Murphy, Ivy Lynn Bourgeault

**Affiliations:** 1 Institute of Management, College of Social Sciences University of the Philippines Baguio, Governor Pack Road, 2600 Baguio, Philippines; 2 Institute of Feminist and Gender Studies, University of Ottawa, 120 Université FSS 11042, Ottawa, Ontario K1N 6N5 Canada; 3 Population Institute, College of Social Sciences and Philosophy University of the Philippines, 1101 Diliman, Quezon City, Philippines; 4 Luke Foundation, Inc., 90 Leonard Wood Road, Engineers’ Hill, 2600 Baguio, Philippines; 5 College of Social Sciences University of the Philippines Baguio, Governor Pack Road, 2600 Baguio, Philippines; 6 Centre for Research on Educational and Community Research, University of Ottawa, 1136 Jean-Jacques Lussier, Ottawa, Ontario K1N 6N5 Canada; 7 Faculty of Medicine, University of Ottawa, 850 Peter Morand Crescent, Ottawa, Ontario K1G 3Z7 Canada; 8 Department of Community Health and Epidemiology, Faculty of Medicine, WHO/PAHO Collaborating Centre on Health Workforce Planning and Research, School of Nursing, Faculty of Health Professions, Dalhousie University, 5869 University Avenue, Halifax, Nova Scotia B3H 4R2 Canada; 9 Telfer School of Management, University of Ottawa, 1 Stewart St., Ottawa, Ontario K1N 6N5 Canada

**Keywords:** Health workers, Migration, Philippines, Human resources for health, Retention, Physicians, Nurses, Midwives, Physiotherapists, Policy

## Abstract

**Background:**

Dramatic increases in the migration of human resources for health (HRH) from developing countries like the Philippines can have consequences on the sustainability of health systems. In this paper, we trace the outflows of HRH from the Philippines, map out its key causes and consequences, and identify relevant policy responses.

**Methods:**

This mixed method study employed a decentered, comparative approach that involved three phases: (a) a scoping review on health workers’ migration of relevant policy documents and academic literature on health workers’ migration from the Philippines; and primary data collection with (b) 37 key stakeholders and (c) household surveys with seven doctors, 329 nurses, 66 midwives, and 18 physical therapists.

**Results:**

Filipino health worker migration is best understood within the context of macro-, meso-, and micro-level factors that are situated within the political, economic, and historical/colonial legacy of the country. Underfunding of the health system and un- or underemployment were push factors for migration, as were concerns for security in the Philippines, the ability to practice to full scope or to have opportunities for career advancement. The migration of health workers has both negative and positive consequences for the Philippine health system and its health workers. Stakeholders focused on issues such as on brain drain, gain, and circulation, and on opportunities for knowledge and technology transfer. Concomitantly, migration has resulted in the loss of investment in human capital. The gap in the supply of health workers has affected the quality of care delivered, especially in rural areas. The opening of overseas opportunities has commercialized health education, compromised its quality, and stripped the country of skilled learning facilitators. The social cost of migration has affected émigrés and their families. At the household level, migration has engendered increased consumerism and materialism and fostered dependency on overseas remittances. Addressing these gaps requires time and resources. At the same time, migration is, however, seen by some as an opportunity for professional growth and enhancement, and as a window for drafting more effective national and inter-country policy responses to HRH mobility.

**Conclusions:**

Unless socioeconomic conditions are improved and health professionals are provided with better incentives, staying in the Philippines will not be a viable option. The massive expansion in education and training designed specifically for outmigration creates a domestic supply of health workers who cannot be absorbed by a system that is underfunded. This results in a paradox of underservice, especially in rural and remote areas, at the same time as underemployment and outmigration. Policy responses to this paradox have not yet been appropriately aligned to capture the multilayered and complex nature of these intersecting phenomena.

## Background

Human resources for health (HRH) are crucial to the achievement of health outcomes and the overall sustainability of health systems [1], and their migration from developing (low- and middle-income) to developed (high-income) countries has raised significant economic and ethical concerns. The Philippines, in common with many other “source” countries, suffers from a high burden of disease and an inequitable distribution of health workers, but has become a key source of health professionals who migrate to wealthier countries [2, 3]. This paper presents the findings of a multi-year mixed method study of source country perspectives on the causes, consequences, and policy responses to the migration of HRH with specific reference to the Philippine context. It addresses one of four countries that participated in the larger study, which also included South Africa [4], Jamaica [5], and India [6], all known for their high rates of HRH migration. The following questions guided the research:

1. What are the recent historic trends and present situation of HRH migration from the Philippines?
2. What, according to the experience of those who remain, are the causes and consequences of the emigration of Filipino HRH?
3. What program and policy responses have been considered, proposed, and implemented by different stakeholders in the Philippines to address these consequences?

### Brief overview of the Philippines context

This Philippine case study on HRH migration situated workforce mobility within the interactions of individual decision-making process and the wider structures of politics, economy, and history of the country. The Philippines is a lower middle-income country in Southeast Asia with a young population of more than 92 million. There are high levels of poverty in the country, with 26.5% of the population living below the poverty line. In the last quarter of the twentieth century, the Philippines, under direction of the International Monetary Fund and the World Bank, implemented a series of structural adjustment measures, modeled on neoliberal economics [7], to ensure the country could repay its foreign debts, largely incurred under the Marcos regime [8]. These conditionalities included *inter alia* reducing spending in health and social sectors, devaluing the currency, and hastening the privatization of public resources, which contributed to increased poverty and economic instability [9, 10]. These policies were continued under the Aquino government and included the automatic appropriation from the annual government budget of the full amount needed for debt servicing [9].

With a median age of 23, the Philippines is a country of young people. Currently, the country is contending with a large number of unemployed and underemployed—an estimated 4.5 and 7.3 million respectively [11]. Over one quarter of the labor force is unemployed or looking for more work [12]. Large numbers of Filipinos, including some of the country’s most educated, are compelled to go abroad to find employment [12]. The Philippines is the second largest exporter of human labor in the world, and health care professionals are one of the biggest groups of migrant labor for the country. The Philippine Overseas Employment Administration (POEA), which facilitates outmigration, reported that 1.8 million Filipinos left the country for work in 2013 [13].

#### The national health system

The Philippines Health Care System, described as decentralized, is organized at three different levels—national, provincial, and local. While health care in the Philippines is provided by both public and private sectors, the total contribution allocated to the public sector has decreased for the past decade. Only 36.1% of the health expenditure is paid by the government; the private sector contributes 65.3% of the total health expenditure, and 83.8% of that private health care expenditure is paid out of pocket. Between 2009 and 2011, government spending on health care averaged 4.3% of GDP, lower than a cited, though never formally adopted, World Health Organization’s (WHO) suggested standard of 5%, illustrating that the country’s healthcare system is underfunded [14]. Per capita health expenditure (at constant 2000 prices) in the country is PHP 2639 or around Php 7 per day, approximately 15 cents USD [14].

### Conceptual framework

Labor migration has widely been viewed as determined by a combination of demographic, socio-cultural, political, and economic factors interacting across macro-, meso-, and micro-levels. Micro-level issues include HRH migrants’ perceptions of their personal and household context in shaping their decision to work overseas. Meso-level phenomena include organizational settings such as workload, working conditions, and career opportunities relative to different health professions, in destination and source countries. These issues are influenced by macro-level phenomena—the myriad political, economic, and social factors at global and national levels including policies and recruitment strategies. Each of these levels intersect with the causes, consequences, and policy responses examined.

## Methods

The study employed a decentered mixed method comparative approach [15], comprising scoping reviews of the literature on health worker migration, surveys of health workers, and interviews with key stakeholders. Researchers in Canada (revealed after review) coordinating the four country studies collaborated with researchers in the Philippines (revealed after review) in undertaking the Philippines component in a way that enhanced comparability. Approval to conduct the study (both survey and interviews) was received from the (revealed after review) Research Ethics Board and from the ethics boards of the (revealed after review).

### Scoping review

The scoping review of the literature followed the process developed by Arksey and O’Malley [16], using the MeSH terms “migration”, “health professionals”, “health worker migration”, “brain drain”, “brain gain”, “return migration”, “health worker exodus”, and “Philippines” in a search of Medline, PubMed, and Embase databases [16]. Sources were included if they addressed the Philippines and were published between 2000 and 2012. This was augmented with in country literature searching in seven university and three organizational libraries. We also searched the gray literature using key public and private stakeholder organizational websites resulting in 20 policy documents. The international research team developed a literature extraction tool to systematically record pertinent aspects of the literature. The literature was analyzed and summarized descriptively, and a preliminary report shared and revised with the Philippines-based research team members.

### Stakeholder interviews

Interviews were conducted with key stakeholders including, but not limited to, professional educators, health profession regulators, national government agency officials who dealt with immigration and HRH, and representatives of local government authorities, private and public sector health facilities, recruitment agencies, migrant advocacy organizations, and professional associations and councils (Table 1). Participants were selected using three criteria: (i) their organization’s active role in social determinants of health and migration-related issues; (ii) their position within the organization (sufficiently senior to speak to the issues); and (iii) their experience related to the research questions we were exploring. The interview guide included a common set of questions asked of all stakeholders, but specific probes were developed to enable targeted data collection.

**Table 1:** Distribution of participants by a stakeholder group

Stakeholder group	Number of participants
Teaching/training institutions	3
Professional regulatory boards	4
Professional/worker association	10
Development partners	5
Recruitment agencies	3
National government agencies	10
Health	(1)
Labor and migration	(8)
Foreign affairs	(1)
Return migrants	2
Total	37

A total of 36 interviews, averaging 45–60 min, were conducted between February 2012 and September 2013. All interviews were digitally recorded, after seeking consent, and transcribed. These data were analyzed simultaneously via systematic, documented procedures of thematic and constant comparative analysis using N-Vivo® 9 software following an initial comparative coding structure that was developed by members of the Canadian team and embellished with emergent codes derived from the Philippines team. This involved an iterative process producing a multifaceted description of the context, policy environment, and experiences of the migration of health care professionals.

### Survey

Building on a common template designed by the international team, two questionnaires were developed involving two modes of data collection—an online version (*n =* 202) and a face-to-face household survey (*n =* 420) administered in Metro Manila and Metro Cebu, the major centers of health services in the Philippines where the chances of reaching respondents who graduated with a health degree would be higher (Table 2). Questions were pre-tested to ensure they could be clearly understood. Both surveys targeted respondents who studied to be physicians, nurses, midwives, and physical/occupational therapists. This paper focuses solely on results of the face-to-face survey. While patterns are generally similar for the face-to-face and online surveys, some are different which necessitates further examination beyond the scope of this overview paper.

**Table 2:** Percent distribution of survey respondents by health profession

Type of respondent	Percent	Number
Doctor	1.7	7
Nurse	78.3	329
Physical therapist/occupational therapist	4.3	18
Midwife	15.7	66
Total	100.0	420

Sampling for the face-to-face household survey used the “30 × 7” cluster sampling technique. Within each of the two metro areas, 30 barangays (villages) were selected with probability proportional to the size of population and seven (7) households were chosen for each selected barangay using the WHO simplified cluster sampling for the Expanded Programme on Immunization (EPI) [17]. All household members who completed formal education or training to become a doctor, a nurse, a midwife, or a physical therapist and who were not necessarily working as health professionals were interviewed. To ensure standardization, interviewers read and recorded responses on the survey instrument. Surveys averaged 60–90 min and were conducted in a time and place deemed appropriate by the respondents. Response rate for the face-to-face household survey was 91%. Data were encoded using CSPro. Data cleaning (for odd codes and consistency) and descriptive analysis using frequency and cross-tabulations were done using SPSS21.

Reflecting the general distribution of health professionals in the country, 78% of the survey respondents are nurses, 16% are midwives, 4% are physical/occupational therapists, and 2% are physicians. Notably, the face-to-face survey is a reflective of perspectives found in Metro Manila and Cebu and may not be representative of the Philippines as a whole.

## Results

### Historic trends and the present picture of HRH migration

The Philippines has been engaged in labor export since the early twentieth century when its colonial rulers, the USA, facilitated the outmigration of agricultural workers to Hawaii and the US mainland [18] and afforded Filipino workers access to the American labor market. Before nursing education was well established in the Philippines in the 1920s–1930s, many Filipino nurses trained in the USA, returning to take up well-respected hospital positions and cementing the notion that going overseas was a pathway to prosperity [18]. In 1974, President Marcos issued Presidential Decree 442 to promote labor export, which led to the development of government agencies dedicated to facilitating outmigration and overseas remittances [18, 19]. The promulgation of labor export policies and programs expanded under the auspices of subsequent Filipino leaders [18]. By the twenty-first century, remittances had become a major source of foreign exchange and constituted a significant part of the Philippine economy—amounting to more than 17% of GDP [19, 20]. Between one third and one half of the Philippine population is dependent on remittances to sustain themselves [21].

Thirty years ago, physicians comprised the major group of health professionals leaving the country. In recent years, however, female health care providers, particularly nurses, have become the dominant migrant group [22]. Annually, 17,000 to 22,000 health professionals leave the Philippines to work abroad [23], most of them nurses who represented 29% of the total number of migrant HRH from 1993 to 2010. In 1998, almost 85% of all nurses were employed overseas compared to only 15% employed in the country [24]. Between 2008 and 2012, 90,382 nurses went to Saudi Arabia, while 15,701 migrated to the UK and 14,895 to the USA [25]. Currently, Singapore and the United Arab Emirates have become major recipients of Filipino nursing personnel. International labor markets have continued to grow, marked by an increase in the number of hired nurses from 11,805 to 17,236 between 2010 and 2011.

### Factors that influence migration

#### Micro-level factors

**Fig. 1:**
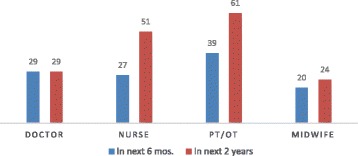
Percentage of health professionals reporting “very likely” to migrate

Stakeholders said that perceptions and evaluations of the country’s situation relative to their personal and family context significantly influenced migration decisions. Survey responses indicated that the desire to migrate was widespread among all health professionals (24% of midwives, 29% of doctors, 51% of nurses, and 61% of PTs in the next 2 years) (Fig. 1). The top four preferred destination countries by survey respondents were the USA, Canada, Australia, and the UK. Among those who said that they contemplated working abroad, 90% indicated that they planned to work as health professionals in the country to which they intend to move.

**Fig. 2:**
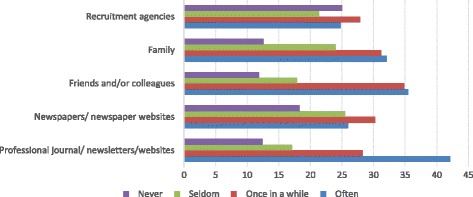
Frequency of seeking migration information by source

In identifying why they wanted to become a health professional, three of the top six reasons cited by survey respondents, perhaps with the exception of physicians, related to the opportunities it provided them to work or migrate overseas (Fig. 2). As a government stakeholder elaborated, however, sometimes the eventual migration of health workers was unrelated to their profession, and instead linked to family members already working overseas:

A lot of those who leave, even though they are graduates of health-related courses, or might be health professionals in the Philippines, do not necessarily apply for visas abroad as workers. It’s just a consequence of them being part of an immigrant family. (Government stakeholder 11272012)

Stakeholders said that perceptions and evaluations of the country’s situation relative to their personal and family context significantly influenced migration decisions. There are also factors that keep some Filipino HRH from migrating overseas, including limited employment overseas, absence of family support, and potential abuse of migrant workers in their destination country. Retaining proximity to their families was also a strong influence on migration decision-making. “There are many stories now about the social cost of the separation of the family due to migration. Some choose not to migrate because they cannot imagine life separate from their family” (Government informant 03182013). Finally, love and passion for, and commitment to, one’s profession were among the reasons many stakeholders gave for staying in the country:

[It’s] because they love the community or [for those] working in the rural communities, providing health services to the community, [it’s] really out of love. (Government informant 11272012)

#### Meso-level factors

Some stakeholders pointed to local organizational settings, specifically dismal working conditions and limited employment or career opportunities, as major factors in migration decision-making.

Are there facilities for health? Are there plantilla^1^ positions for health, for all these professionals, for them to engage not only in gainful employment but to practice their profession? (Government informant 07152013)

**Fig. 3:**
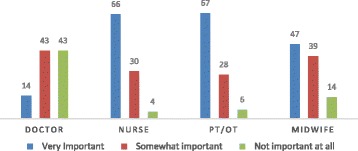
Level of job satisfaction in migration decision-making among survey respondents

For a majority of survey respondents, job satisfaction was a crucial factor in migration decisions (Fig. 3). The level of satisfaction among different health worker groups is reasonably high for respect but is low for income and ambivalent with work benefits, workload, and infrastructure.

Stakeholders noted how poor funding of the healthcare system, resulting in unemployment or underemployment, compelled many health workers to seek work overseas. Across health professions, career opportunities in the Philippines are also limited: “There are not enough training opportunities for doctors to specialize” (Government informant 07152013) and “nurses are not able to practice their profession because of the lack of positions in hospitals” (Recruitment agency informant 06112013). Some informants noted that an ongoing privatization of public health institutions has led to HRH job loss, compelling workers to seek opportunities overseas: “the government’s existing policy is to privatize [the health system]. And with privatization comes more push for migration” (Migrant advocacy group informant 01222013). Some stakeholders also noted that the country’s health education curriculum pushes health workers to migrate:

What is the content of your curriculum? Do you expose them to the hard realities of life, or do you limit them to tertiary hospitals? What kind of exposure do they get? Do they have exposure to public health? (Training institute informant 04052013)

Participants across stakeholder groups agreed that better salaries and working conditions entice Filipino HRH overseas.

The problem is, they have a low salary [here] and…you’ll be working for a lot of bosses since you work for the local government. You’ll be subjected to a lot of politics. (Migrant advocacy group informant 01222013).

Furthermore, one nurse leader noted that Filipino health workers are pulled towards overseas employment because of the quality of practice and the respect given to health professionals. Other important factors included opportunities for advanced professional learning and training overseas.

Finally, some health professionals also find that they cannot utilize their skills in the Philippines due to what are perceived as outdated practice guidelines. Midwifery regulations, for example, limit “the practice of midwifery only to low-risk deliveries [while] internationally, midwives can do other high-risk functions” (Training institute informant 08062013). As with other health professions, such practice scope limits may propel midwives to seek foreign employment where more of their training and knowledge can be deployed.

#### Macro-level factors

Our interviews demonstrated that migration and its causes are disputed phenomena. Participants from government agencies explain that labor movement is part of every citizen’s right to mobility. One stated that: “migration is the stepchild of globalization… we cannot prevent the professionals from going out” (Government informant 07152013).

Some stakeholders were optimistic that migration is a key to solving the country’s unemployment problem and oversupply of nurses. Some nursing leaders argued that migration is an opportunity for social mobility and for Filipino HRH to prove their capacity to “nurse the world.” There are respondents who view migration as a temporary outflow, a positive situation, which, when facilitated well, could lead to better things: “I think we should not stop (migration). We should give more consideration for people going abroad and we should not make it hard for them” (Professional association informant 03182013).

Advocacy group representatives, in sharp contrast, argue that labor migration is forced mobility rooted in limited opportunities in the country. Some stakeholders shared the view that migration and the orientation of Filipino graduates to work overseas was the result of colonization, media hype, and the proliferation of a discourse of “migration as a sign of success” (Government informant 03132013).

In addition, the national economic and political situation impacts HRH migration.

They [HRH] see the Philippines as not being the most conducive place for their family, and being parents, you want to provide the best opportunity for your children. So naturally, people would want to migrate instead. (Recruitment agency 06112013)

Militarization and branding of health workers as anti-state activists further incentivizes HRH migration [26]. The militarization and safety concerns in some rural areas have led to extreme measures:

The doctors bring a gun instead of a stethoscope… and they are trained to shoot guys. In the south^2^ for example, if you’re living in the city and there are three hospitals that you cover and you have to travel at night. You have to be ready for (security) emergencies. (Professional association informant 02282013)

Finally, it was argued that the forces of neoliberal globalization opened the arena for recruitment of migrant health workers as a cost-efficient economic strategy for both the Philippines and destination countries.

The Philippines has the best system for migration… (the government) has these machinery or system created to facilitate migration POEA [Philippines Overseas Employment Administration], DOLE [Department of Labor and Employment], OWWA [Overseas Workers’ Welfare Administration]… We are the role model. Remittances are used to pay debt. … Migration is our best industry. (Migrant advocacy group informant 01162013)

On the other hand, countries with less stringent policies on immigration and employment greatly influence migration decisions. Canadian and American policies and practices facilitated the recruitment of HRH to North America. The Middle East, prior to the revisions of employment rules and Saudization which is meant to reduce reliance on foreign labor and enhance employment opportunities for Saudi citizens, was also a top destination as countries in the region did not require additional licensure and training. Government-to-government recruitment or bilateral agreements (i.e., Germany, UK, and some regions of Canada) also played significant roles in the institutionalization of Filipino HRH’s overseas migration.

These countries have also become top destinations for Filipino migrant HRH because of opportunities for HRH’s families to immigrate: “If you go to Canada, you go to the US, your family is automatically included into the petition, so (it is among) some of the factors” (Recruitment agency informant 05092013). As noted in the survey findings, English speaking countries remain among the top destinations of Filipino health professionals. For countries that demand proficiency in other languages for employment, the availability of training institutes and the guarantee of remuneration equivalent to local HRH (e.g., Germany) serve as pull factors for Filipino HRH.

### Consequences of migration

The migration of health professionals has had important consequences for the health system in the Philippines, affecting both health professionals and the population in need of their services. Stakeholders argued that it aggravated the existing deficit for specialized health professionals and the inequitable production and distribution of health workers across the country. One asked: “How do you respond to the health needs of the country when those who can respond are outside of the country?” (Migrant advocacy group informant 01162013).

The survey results suggested that shortages of workers and reduction in services provided were the most evident effects of health worker migration according to survey respondents (Table 3). Respondents also highlighted increased errors and poorer quality of health care service provision as results of the continuing HRH migration.

**Table 3:** Impact of health workers’ migration from the Philippines

Impact (first ranked)	Doctor	Nurse	PT/OT	Midwife	All
Shortages of workers	71.4	41.6	55.6	33.3	41.4
Increased over time	0.0	11.2	0.0	13.6	11.0
Reduction in services being provided/required	14.3	21.6	5.6	18.2	20.2
Increases errors/poorer quality of care being delivered	0.0	8.5	5.6	7.6	8.1
Patients/clients reporting problems accessing services	14.3	3.3	0.0	9.1	4.3
Longer waiting times	0.0	7.3	5.6	7.6	7.1
Have noticed no (other) impacts	0.0	0.6	5.6	0.0	0.7
Other	0.0	0.9	0.0	0.0	0.7
No information	0.0	4.9	22.2	10.6	6.4

Stakeholders argued that HRH migration negatively affected the functioning of the health care system as generally health professionals with substantial amounts of training, experience, and skills are the ones who leave.

We have nurses who are very good. Where are they now? One left for US, two left for Australia, and there are the very good faculty that we are hoping to be our replacement someday. Now they’re gone (Professional association informant 07152013).

Nevertheless, there are some respondents who expressed wariness on the perception of a “brain drain” caused by HRH migration.

Are you saying that those who out are there have brains, but those who are left behind have none?… let’s just put things in the proper perspective (Government informant 07152013).

Stakeholders from government institutions, professional associations, and training institutes argued that return migration has the capacity to bring about knowledge transfer and improvements in health care practice in the country. We found, however, among our survey respondents that typically, Filipino labor migrants would want to return to the Philippines when they can no longer work (Table 6). Specifically, the majority of survey respondents would want to retire in the Philippines and be buried in the Philippines (Table 4).

**Table 4:** Survey responses to return migration by profession

Proportion* who would…	Doctor	Nurse	PT/OT	Midwife	All
Want to retire in the Philippines	100.0	80.3	93.8	75.0	80.5
Want to be buried in the Philippines	100.0	94.1	100.0	97.2	94.9
Number of cases	2^a^	239	16	36	293

*Among those who responded they have given much consideration to moving to another country to live and work, and are likely or very likely to leave in six months to two years

^a^The small sample of doctors means that this finding should be treated with caution

In addition, the projection of available opportunities for health professionals also influenced the country’s health education system. All key informant groups acknowledge that the most obvious impact of migration had been the uncontrolled expansion of certain health professions, in particular, nursing education.

I think the problem started late 90s to early 2000s because of the increased enrollment in nursing. As of now there are 491 colleges, and before it was only half. And so I think because of the demand, they all got attracted to go into nursing, and the main reason during that time, when it was still early, was to go abroad. (Training institute informant 08052013)

Respondents argued that this expansion also led to the commercialization and deterioration of nursing education standards. Moreover, the burgeoning numbers of nursing graduates cannot be absorbed by the health care system. Resultantly, nurse unemployment and underemployment contributes to increased outmigration. Health institutions face greater costs to constantly recruit, train, and retrain personnel to fill positions left vacant by emigrating HRH. The delivery of health care services in rural underprivileged areas of the country has been greatly affected by the perennial departure of health professionals.

There’s inadequacy of health workers because most of the health workers in the public sectors would go into the urban areas. Some of rural health workers would end up working overseas.... Barangay [village level] officials would appoint Barangay health workers who are trained but not necessarily about the nursing profession, but more on first aid…so that’s the reason why health services in the rural areas are in dire (condition) (Government informant 11272012).

#### Consequences for Filipino migrant health workers

Most respondents recognized that overseas migration may have a positive impact on individual Filipino health workers; however, there were some adverse effects as well. Key stakeholders from all sectors noted that migrant Filipino HRH experience exploitation, discrimination, unfavorable work load, and human rights violations.

One form of exploitation is on hours of work. The Filipino workers are not given what is due them. What is agreed upon before departure for the destination country, the salary agreed upon is not given to them. And then the inhuman conditions, the treatment. That is the most common (Government informant 03262013).

In addition, migrant Filipino health workers also experienced and suffered from illegal recruitment, contractualization (a government system which allows a company or an employer to hire a worker for a temporary period without security of tenure or benefits) and contract substitution.

There is a case of doctors who have migrated abroad in the United States but work there as medical representatives of drug companies or as nurses…There are nurses who migrated but are employed as a private nurse and are treated like a domestic helper (Government informant 03182013).

#### The consequences on migrant households in the context of the national economy

The contribution of migrant remittances to the economic development of source countries has been a key reason why governments support labor export [10, 27]. Remittances may have kept the Philippine economy afloat since the formalization of its labor export policies under Marcos, and it has been a concrete survival mechanism for many families. Survey respondents who are considering migration within the next 6 months to 2 years affirmed that they would send remittances to the Philippines (Table 5).

**Table 5:** Survey responses to post migration activities by profession

Proportion* who would…	Doctor	Nurse	PT/OT	Midwife	All
Send money home	100.0	98.7	100.0	100.0	99.0
Want to become a permanent resident of this country?	100.0	62.3	56.3	61.1	62.1
Want to become a citizen of this country?	100.0	65.3	62.5	61.1	64.8
Number of cases	2^a^	239	16	36	293

^a^The small sample of doctors means that this finding should be treated with caution, although an online survey (not reported on in this article) had a sample of 13 doctors, 85% of whom indicated that they would send money home

*Among those who responded they have given much consideration to moving to another country to live and work, and are likely or very likely to leave in six months to two years

Remittances may have a direct impact on migrants’ families and indirectly to the economy: “When the family procures things that they don’t normally buy without the remittances, and then a certain amount of money goes to the government by way of the Value Added Tax” (Professional association informant 07152013). Another respondent remarked that remittances affect the economy in the form of human capital development when migrants’ families invest in education.

… An OFW [overseas Filipino worker] who can afford to send the children to good schools would certainly have good qualified children that will pursue employment in the future both locally and overseas. A global workforce is easily achievable. (Government informant 03182013)

Some stakeholders noted the exponential growth of remittances in the country over recent years but acknowledged that the contribution of labor migration to the country’s economy cannot be accurately measured. Importantly, the accrual of education does not always translate into remunerative employment as evidenced by the under- and unemployment of professionals including HRH and the numbers of educated Filipinos who work abroad as household service workers [28].

Additionally, participants considered the impact of migration on families. One concern was familial overdependence on, and misspending of remittances. “There are also stories where if the OFW is able to send home lots of money, his or her family in the Philippines stops working and relies solely on the OFW” (Professional association informant 03182013). Migration may also lead to the deterioration of familial relationships or family disintegration.

### Policy responses

Policy and program responses managing HRH migration span international, national, and regional levels. These responses include (1) the enactment of retention and reintegration programs; (2) the signing of multilateral and bilateral agreements and recruitment codes; (3) the establishment of international organizations attempting to document migration flows and advise on policies to mitigate negative externalities; and (4) the promotion of initiatives towards the protection of migrant workers’ welfare and incentives for returning émigrés. The impact of these policies depends on the form and scope of the policy proposed and implemented, the extent of their enforcement and reach, and their transferability across different levels of management and regulation.

#### Retention and reintegration programs

Some stakeholders offered recommendations concerning the retention of health care workers in the country, which address the perceived causes of Filipino HRH migration. These include short- and long-term policy solutions such as improvements in working conditions, providing financial and technical support for the health sector, improving health education curricula, and establishing good governance practices.

How much do health workers get for their salary? … Before we can even have a promise of a retention policy, these things need to be take care of (Government informant 07152013)

A policy of good governance that will cover nationalism and patriotism. Good governance is a good intervention that I think will prevent migration of our nursing—and other health—professionals (TI Informant 04052013).

Some respondents also noted that the Magna Carta for Public Health Workers of 1992 (MCPHW) should be properly implemented. Through the provision of improved salaries and compensation, the MCPHW was meant to increase the number of health workers serving in marginalized areas, although “it is not being observed, the Magna Carta is not being implemented” (Government informant 08122013). In addition, the devolution of public health services in 1995 found that some local governments were ill-equipped to implement the law.

To address the problem of uneven distribution of healthcare professionals among urban and rural areas, the Philippine government implemented a program to train medical and nursing graduates to serve in rural or underdeveloped areas, which included a return-of-service requirement. More than three fourths of survey respondents thought it was justified for all health professionals trained in the country to do return of service or community work for a specified time, and 87% agreed that this was especially true for those who received government scholarships (Table 6).

**Table 6:** Survey respondents who feel that the Philippines government is justified requiring return of service

Possible requirements	Percent
All health professionals trained in the country to do return of service/community service for a specified amount of time	76.7
All health professionals who have received government scholarship for education to complete some form of national service	86.9
All health professionals trained in the country to work in a rural or underdeveloped area for a specified amount of time	67.4
All health professionals who have received government scholarship to work in a rural or under-served area for a specified amount of time	81.2

#### Multilateral and bilateral agreements

To directly address practices and policies on recruitment of HRH from under-resourced countries such as the Philippines, international and multilateral ethical codes of conduct have been developed [29, 30]. Bilateral agreements serve as a method of regulating HRH migration in number, type, and quality of health personnel recruited from source to destination countries with the aim that both countries derive benefits from the exchange, while protecting the welfare and rights of the migrating health workers [27]. To this end, the Philippine government has undertaken arrangements with partnering countries in the formulation and implementation of bilateral agreements on the flow of HRH (see Table 7). Nevertheless, to harness the benefits of knowledge and technology transfer, some stakeholders recommended that provisions in bilateral agreements should allow willing Filipino health professionals to return for 1 or 2 months to share their skills and talents gained from experience with the destination country without risk of job loss.

**Table 7:** Bilateral labour agreements between the Philippines and other countries

Middle Eastern countries: -Saudia Arabia—no permanent residency, employers hold passports, meals are provided, living stations as well. Workers can only work from Saturday to Wednesday, 8 h a day or 48 h a week. At least one rest day per week, overtime pay during Fridays, 30 days leave per 2 years, transport allowance if workers live far from work; as prescribed by the laws of the Kingdom of Saudi Arabia and the accord entered with the Philippine government. -United Arab Emirates—no permanent residency, although all nationalities are treated equally. Violence, restriction of physical movement, and economic exploitation are disputed. -Kuwait—no permanent residency but is reportedly the most “Filipino friendly” among the three Arab nations reported [28].
Canada: From 2007 to 2008, the Philippines signed a bilateral agreement with the Canadian Provinces of Saskatchewan, Manitoba, and Alberta. These agreements incorporated new targeted migrant schemes to speed up the process of getting workers to where they are needed while securing their rights and welfare [55]. In addition, by entering into these agreements, the Philippine government has aimed to ensure an ethical recruitment of health professionals that does not deplete their healthcare labor force. For instance, in the country’s agreement with Saskatchewan, it has been agreed that for every 10 nurses, the importing country will improve the nursing center in the Philippines and for one Filipino nurse hired, three more nurses will be educated. Joint research, linkages, and graduate scholarships with their universities for improved research were also included in the agreement [56].
European countries: Switzerland involves the exchange of professionals and technical trainees for short-term employment; the agreement with the UK aims to help the recruitment of Filipino health professionals; and the Philippines-Norway agreement aims to develop cooperation to reduce the demand for professionals in the health sector in Norway and to promote employment opportunities for Filipino health personnel [57].

For most of the stakeholders, however, these actions and policies have neither restricted nor effectively regulated the migration of Filipino health workers; rather, they have systematized further their recruitment and mobility. Stakeholders’ perspectives revealed that because the adoption of international recruitment codes is only voluntary, they remain considerably ineffective in the management of international migration of HRH [31]. Despite the presence of these agreements, some participants highlighted how the Philippines has faced significant setbacks, owing to disagreements regarding the equivalence of education, training, and quality of the country’s HRH.

## Discussion

Individual decision-making about migration must be situated within a broader socioeconomic, historical context that attends to both discursive and material aspects of power operating across individual, institutional, national, and global spheres. At the micro-level, individual reflections on the country’s political and economic situation and the desire for career advancement influence the decision to migrate. These findings echo the results of previous research [32–40]. Individuals may feel pushed to migrate due to the poor wages offered to HRH and pulled to migrate by the prospect of better social, economic, and professional opportunities abroad and by the presence of overseas kin. Countering these elements are ones that encourage health professionals to remain in the Philippines including dedication to family, culture, and community, and concern about encountering cultural differences, discrimination and workplace abuse, loss of social support, and the negative impact of family separation. The presence of family and the enhanced respect respondents anticipated they would receive as health professionals in another country could increase the likelihood that they would remain overseas.

At the meso-level, better economic benefits and organizational settings were seen as factors that affect migration decisions. The literature points to risks of work-related hazards [36], and inadequate health care system and shortages in human resources [22, 41, 42] as the primary meso-level factors that influence the individual’s decision to migrate. Specifically, poor health care infrastructure, low wages, job insecurity, inconsistencies in practice, outdated or inappropriate curricula, institutional politics, and inadequate opportunities for speciality training were all cited as influencing migration decisions while return community service and improved curricula were would encourage health workers to remain. Concomitantly, the potential to engage in advanced training and the perception of greater equality among, and respect for, health professionals overseas were regarded migration incentives. Interestingly, nearly three quarters of respondents chose a health career because of the potential for overseas opportunities. Importantly, the increasing number of trained nurses due to the rapid expansion of nursing programs, who had hoped to train graduates destined for the overseas market were confronted by a stagnant global market resulting in increased under- and unemployment. Many trained professionals were therefore compelled to take up lower skilled positions abroad, thereby thrusting them onto a path of deskilling [43, 44]. Complicating the scenario is the declining quality of nursing education, which previous research suggests is the consequence of the commercial expansion of nursing programs whose goal is to produce graduates for export [22, 42, 45–47], and an oversupply of health workers who cannot secure positions in the underfunded Philippine system.

Certain issues such as privatization of health care services and the prominent discourse of migration as key to success were situated at the interface of meso- and macro-level analyses. The impact of neoliberal globalization (structural adjustment and, more recently, post-financial crisis austerity programs, [c.f. 7]) has engendered the withdrawal of state support for health, social services, and education and promoted privatization contributing to job insecurity and unemployment. Labor export policies and programs provide an avenue for unemployed and under-employed, and encourage remittances from overseas Filipino workers. Ongoing state-supported human rights abuses, particularly in rural regions, also propel the exodus of health professionals and reinforce the maldistribution of health resources. Informed by discourses that have normalized migration and emphasized the right of citizens to migrate, HRH are attracted to overseas work by specific destination country policies, government-to-government agreements, and recruitment agency activities. These observations are corroborated by previous studies that enumerated the “culture of migration” [48], labor export policy [22, 48, 49], and unemployment [41, 42, 50] as major push factors at the macro-level. Stakeholders further identified the development of specific retention programs, the implementation of the Magna Carta for Health Workers, and improved infrastructure as policies that could encourage HRH to remain in the Philippines, while the ability to settle abroad with one’s family and to return temporarily to contribute to knowledge and technology transfer were regarded as factors that would inspire HRH to remain overseas.

HRH migration impacts individuals, families, institutions, services, society, and nation states. While some key stakeholders argued that return migration or “brain circulation” offered benefits through short- or long-term knowledge transfer and exchange, there is insufficient evidence in the literature to support these assertions. Country case study findings on brain drain are consistent with the literature describing it as a process manifested in the deficit of specialized health professionals with grave consequences to both the health care system and the individual health workers [41, 51]. The migration of health professionals results in a paucity of skilled personnel in health institutions across the country, especially in rural areas. HRH mobility also burdens the remaining health workers in the country in terms of workload. Previous studies suggest that if this migration trend continued, the Philippine health care system would be severely disadvantaged or worse, it would collapse [38, 41, 52–54].

The plight of an individual HRH cannot be separated from the existing condition of the local health system and the country itself. The opportunities for professional and personal growth, economic wellbeing, and the conditions of local practice interact with the existing policies and programs of the country. While these affect the retention or dissatisfaction of HRH, the country’s health sector financing and health workforce management schemes are also inseparable from international political and economic conditions. Reductions in government expenditures in health, privatization as health financing strategy, and rationalization in public health institutions as a workforce management scheme are not de facto conditions present in the national health system. These are historical products of programs and policies instituted by the government and which include economic liberalization, structural adjustment programs, and the Labor Export Policy.

## Conclusions

Grounded in a colonial legacy that has normalized labor migration as a means of social and economic mobility and propelled by the exigencies of neoliberal globalization, the Philippines has developed a sophisticated state apparatus that facilitates migration and encourages OFW remittances. HRH comprise an important sector of labor migrant flows and as such the Philippines continues to produce doctors, nurses, midwives, and other health professionals who are highly specialized and sought after in countries across the globe. The country’s dominance as a HRH exporter, however, means that it is losing its skilled resources while struggling to manage its own health care services, particularly in under-served, rural areas. With HRH migration, the Philippines is at the disadvantage not only due to the creation of a workforce predisposed for overseas employment instead of serving locally but also through the loss of a skilled workforce that is in essence given away to the benefit of destination countries. While host countries benefit from the care provided by the migrant Filipino health professionals, many of these health professionals are also subject to discrimination, exploitation, wage differentials, and deskilling. As the HRH work their way to provide for their families in the Philippines, the societal cost also can often outweigh the personal benefits of migration. Despite the drawbacks, the massive expansion in education and training designed specifically for outmigration creates a domestic supply of health workers who are not being absorbed locally despite high needs especially in rural and remote areas. Although the majority of trained health professionals remain in the country, the numerous interacting micro-, meso-, and macro-level factors that propel Filipino HRH to seek work overseas far outweigh the factors that foster retention at home. International agreements, ethical recruitment guidelines, and programs to protect overseas HRH may mitigate some of the more egregious forms of exploitation they face; however, the complex and sometimes paradoxical nature of these intersecting, multi-level phenomena and their consequences will require greater consideration and systemic change. Importantly, major financial investments in health, education, and social services, and greater control over public resources are required to redress social and economic inequalities and the deteriorating human rights situation that contributes to the loss of HRH to ensure that migration is truly a choice for health professionals—one that Filipinos across the archipelago can afford.

## Abbreviations

ASEAN: Association of Southeast Asian Nations
CEO: Chief executive officer
CSPro: Census and Survey Processing System (software)
DOH: Department of Health
DOLE: Department of Labor and Employment
GDP: Gross Domestic Product
HRH: Human resources for health
LEP: Labor Export Policy
MeSH: Medical subject headings
MHO: Municipal Health Officer
NSCB: (National Statistics Coordination Board) Philippine Statistics Authority
OFWs: Overseas Filipino workers
OWWA: Overseas Workers Welfare Administration
PHP: Philippine peso
POEA: Philippine Overseas Employment Administration
PT/OT: Physical therapists (physiotherapists) and occupational therapists
SAR: Special Administrative Region (Hong Kong)
SPSS: Statistical Package for the Social Sciences (software)
USA: The United States of America
US: United States
USD: US dollar ($)
WHO: World Health Organization

## Footnotes

1 In the Philippines, a plantilla refers to an approved listing of personnel for a particular government agency; someone in a plantilla position is regarded as a regular employee.

2 Referring to southern Philippines, which include the island groups of Visayas and Mindanao.

## References

[1] WHO (World Health Organization) (2006). Working together for health–2006.

[2] Mackey TK, Liang AA (2012). Rebalancing brain drain: exploring resource reallocation to address health worker migration and promote global health. Health Policy.

[3] O’Brien P, Gostin LO. Health worker shortages and global justice. Milbank Memorial Fund. 2011. [Internet]. Available from: http://www.milbank.org/publications/milbank-reports/158-reports-health-worker-shortages-and-global-justice-2.

[4] Labonté R, Sanders D, Mathole T, Crush J, Chikanda A, Dambisya Y, Runnels V, Packer C, McKenzie A, Tomblin Murphy G, Bourgeault IL (2015). Health worker migration from South Africa: causes, consequences and policy responses. Hum Resour Health.

[5] Tomblin Murphy G, MacKenzie A. Waysome B, Guy-Walker J, Palmer R, Elliott RA, Rigby J, Labonté R, Bourgeault IL. A mixed-methods study of health worker migration from Jamaica. Hum Resour Health. 2016; 14 (Sup 1). https://human-resources-health.biomedcentral.com/articles/10.1186/s12960-016-0125-8 10.1186/s12960-016-0125-8 PMC4943490 27380830

[6] Walton Roberts, M, Runnels V, Rajan I, Sood A, Thomas P, Nair S, Packer C, MacKenzie A, Labonté R, Tomblin Murphy G, Bourgeault IL. Source country perspectives on the migration of highly trained health personnel: Causes, consequences and responses in India. Hum Resour Health. (in press).

[7] Labonté R, Stuckler D (2016). The rise of neoliberalism: how bad economics imperils health and what to do about it. J Epi Com Health.

[8] Diokno-Pascual MT (2000). The turbulent and dismal record of World Bank structural adjustment lending in the Philippines.

[9] Bello W (2010). The food wars.

[10] Spitzer DL, Piper N (2014). Retrenched and returned: Filipino migrant workers during times of crisis. Sociology.

[11] IBON Foundation (2014). Confronting underdevelopment through people’s struggles.

[12] IBON Foundation Inc (2014). Economy under the Aquino administration: a case of worsening exclusivity.

[13] POEA (2014). Overseas Philippine Worker Statistics.

[14] Asia Pacific Observatory on Health Systems and Policies. Philippines Living Hit Update. *Health Systems in Transition*, September 2013. 2013; Available from: http://www.wpro.who.int/asia_pacific_observatory/hits/series/phl_living_hits_3_2_health_expenditure.pdf.

[15] Wrede S, Benoit C, Bourgeault IL, Sandall J, DeVries R, Van Tiejlinden E (2006). Decentred comparative research: context sensitive analysis of health care. Soc Sci Med.

[16] Arksey H, O’Malley L (2005). Scoping studies: towards a methodological framework. Int J Soc Res Methodol.

[17] Henderson RH, Sundaresan T (1982). Cluster sampling to assess immunization coverage: a review of experience with a simplified sampling method. Bull of WHO.

[18] Rodriguez RM (2010). Migrants for export: how the Philippine state brokers labor to the world.

[19] Barber PG, Tastsoglou E, Dobrowlsky A (2006). Locating gendered subjects in vocabularies of citizenship. Women Migr. Citizsh. Mak. Local Natl. Transnatl. Connect.

[20] Soriano L, Ofreneo R, Samonte I, Soriano L, Prieto P (2009). Remittances and the Philippine mal-development. Migr. Dev. Policy Reforms.

[21] Parreñas RS (2005). Children of global migration: transnational families and gendered woes.

[22] Brush BL, Sochalski J (2007). International nurse migration: lessons from the Philippines. Policy Polit Nurs Pract.

[23] WHO 2013 World Health Organization (2013) World Health Statistics 2013 Geneva: World Health Organization. Retrieved from http://www.who.int/gho/publications/world_health_statistics/EN_WHS2013_Full.pdf.

[24] Lorenzo FM, de la Rosa J, Cella V, Celino S (2011). National profile of migration of health professionals—Philippines [Internet].

[25] POEA (2012). 2008–2012 Overseas Philippine Worker Statistics.

[26] Boehringer GH. (2012) Blighted: Philippine Jurisprudence and State Repression – the “Morong” 43. http://bulatlat.com/main/2010/04/12/blighted-philippine-jurisprudence-and-state-repression-the-morong-43/ (cached: http://www.arkibongbayan.org/2010/2010-04April18-MorongDocs/docendre/05-Morong%2043%20BLIGHTED%20Boehringer%20article.doc.

[27] International Labour Organization (ILO) (2009). Protecting the rights of migrant workers: a shared responsibility.

[28] Cruz GT, Laguna EP, Arifin EN, Ananta A (2009). Overseas labour migration and well-being of older Filipinos. Older persons in SE Asia: an emerging asset.

[29] Connell J, Buchan J (2011). The impossible dream? Codes of practice and the international migration of skilled health workers. World Med Health Policy.

[30] Runnels V, Labonté R, Packer C. Reflections on the ethics of recruiting foreign-trained human resources for health. Hum Resour Health Electron Resour. 2011;9. Available from: https://human-resources-health.biomedcentral.com/articles/10.1186/1478-4491-9-2; doi 10.1186/1478-4491-9-2. 10.1186/1478-4491-9-2 PMC3032656 21251293

[31] Bourgeault IL. Labonté R, Packer C, Runnels, V, Tomblin Murphy G. Knowledge and potential impact of the WHO Global Code of Practice on the International Recruitment of Health Personnel: Does it matter for source and destination country stakeholders? Hum. Resour. Health – special supplement on the WHO Code. 2016; 14 (Sup 1). https://human-resources-health.biomedcentral.com/articles/10.1186/s12960-016-0128-5 10.1186/s12960-016-0128-5 PMC4943484 27381004

[32] Henderson L, Tulloch J (2008). Incentives for retaining and motivating health workers in Pacific and Asian countries. Hum Resour Health.

[33] Allan H, Larsen JA (2003). “We need respect”: experiences of internationally recruited nurses in the UK [Internet].

[34] Daniel P, Chamberlain A, Gordon F. Expectations and experiences of newly recruited Filipino nurses. Br J Nurs. 2001;10(4):254–6, 258–65. 10.12968/bjon.2001.10.4.5374 12170651

[35] Lorenzo M (2002). Nurse supply and demand in the Philippines.

[36] Brown RPC, Connell J (2004). The migration of doctors and nurses from South Pacific island nations. Soc Sci Med.

[37] Lorenzo F, Dela Rosa J, Yabes J (2005). Philippines HRH Master Plan 2005–2030.

[38] Clark PF, Stewart JB, Clark DA (2006). The globalization of the labour market for health-care professionals. Int Labour Rev.

[39] Alonso-Garbayo A, Maben J (2009). Internationally recruited nurses from India and the Philippines in the United Kingdom: the decision to emigrate. Hum Resour Health.

[40] Hayne AN, Gerhardt C, Davis J (2009). Filipino nurses in the United States: recruitment, retention, occupational stress, and job satisfaction. J Transcult Nurs.

[41] Lorenzo FM, Galvez-Tan J, Icamina K, Javier L (2007). Nurse migration from a source country perspective: Philippine country case study. Health Serv Res.

[42] Masselink LE, Lee S-YD (2010). Nurses, Inc.: expansion and commercialization of nursing education in the Philippines. Soc Sci Med.

[43] Pratt G. From registered nurse to registered nanny: Discursive geographies of Filipina domestic workers in Vancouver, B.C. “In Reading Economic Geography, T.J. Barnes (ed). Malden, MA: Blackwell Publishing. Pp. 375–388. In: Barnes TJ, Peck J, Sheppard E, Tickell A, editors. Read. Econ. Geogr. Malden, MA: Blackwell Publishing; 2003. p. 375–88.

[44] Salami B, Nelson S (2014). The downward occupational mobility of internationally educated nurses to domestic workers. Nurs Inq.

[45] Goode AS (2009). Global economic changes and the commodification of human capital: implications of Filipino nurse migration. East Asia.

[46] Cheng MH (2009). The Philippines’ health worker exodus. Lancet.

[47] Villegas MCL (2012). Achievement tests and their relevance to examination performance of nursing graduates. IAMURE Int J Health Educ.

[48] Dimaya RM, McEwen MK, Curry LA, Bradley EH. Managing health worker migration: A qualitative study of the Philippine response to nurse brain drain. Hum Resour Health. 2012;10. alternative: https://www.ncbi.nlm.nih.gov/pmc/articles/PMC3541120/. 10.1186/1478-4491-10-47 PMC3541120 23249411

[49] Hawthorne L (2001). The globalisation of the nursing workforce: barriers confronting overseas qualified nurses in Australia. Nurs Inq.

[50] Ronquillo K, Elegado-Lorenzo FM, Nodora R (2005). Human resources for health migration in the Philippines: a case study and policy directions.

[51] Lorenzo FM, Dela Rosa Jannifer F, Villegas S, Yabes J, Trinidad F, Fernando G (2006). Migration of health workers: country case study Philippines [Internet]. International Labour Office.

[52] Troy PH, Wyness LA, McAuliffe E. Nurses’ experiences of recruitment and migration from developing countries: a phenomenological approach. Hum Resour Health. 2007;5. Available from: https://human-resources-health.biomedcentral.com/articles/10.1186/1478-4491-5-15. 10.1186/1478-4491-5-15 PMC1894817 17555575

[53] Anderson BA, Isaacs AA (2007). Simply not there: the impact of international migration of nurses and midwives—perspectives from Guyana. J Midwifery Womens Health.

[54] Labarda MP. Career shift phenomenon among doctors in Tacloban City, Philippines: lessons for retention of health workers in developing countries. Asia Pac Fam Med. 2011;10. Available from: https://www.ncbi.nlm.nih.gov/pmc/articles/PMC3204289/. 10.1186/1447-056X-10-13 PMC3204289 21977902

[55] Blank NR (2011). Making migration policy: reflections on the Philippines’ bilateral labor agreements. Asian Polit Policy.

[56] Sabater MR (2009). Win-win bilateral agreements to ensure ethical recruitment of Pinoy health professionals abroad urged.

[57] Orbeta A, Abrigo M. 2011. Managing International Labour Migration: The Philippine Experience. Philippine Institute of Development Studies Discussion Paper Series 2011-33. Available from: http://dirp3.pids.gov.ph/ris/dps/pidsdps1133.pdf.

